# Comparing Natural and Constrained Movements: New Insights into the Visuomotor Control of Grasping

**DOI:** 10.1371/journal.pone.0001108

**Published:** 2007-10-31

**Authors:** Chiara Begliomini, Andrea Caria, Wolfgang Grodd, Umberto Castiello

**Affiliations:** 1 Centre for Mind and Brain Sciences, University of Trento, Rovereto, Italy; 2 Institute for Medical Psychology, Eberhard-Karls University of Tuebingen, Tuebingen, Germany; 3 Section Experimental MRI of the CNS, Department of Neuroradiology, Tuebingen University Hospital, Tuebingen, Germany; 4 Department of General Psychology, University of Padua, Padua, Italy; 5 Department of Psychology, Royal Holloway, University of London, Egham, United Kingdom; Harvard Medical School, United States of America

## Abstract

**Background:**

Neurophysiological studies showed that in macaques, grasp-related sensorimotor transformations are accomplished in a circuit connecting the anterior intraparietal sulcus (area AIP) with premotor area F5. Single unit recordings of macaque indicate that activity of neurons in this circuit is not simply linked to any particular object. Instead, responses correspond to the final hand configuration used to grasp the object. Although a human homologue of such a circuit has been identified, its role in planning and controlling different grasp configurations has not been decisively shown. We used functional magnetic resonance imaging to explicitly test whether activity within this network varies depending on the congruency between the adopted grasp and the grasp called by the stimulus.

**Methodology/Principal Findings:**

Subjects were requested to reach towards and grasp a small or a large stimulus naturally (i.e., precision grip, involving the opposition of index finger and thumb, for a small size stimulus and a whole hand grasp for a larger stimulus) or with an constrained grasp (i.e., a precision grip for a large stimulus and a whole hand grasp for a small stimulus). The human anterior intraparietal sulcus (hAIPS) was more active for precise grasping than for whole hand grasp independently of stimulus size. Conversely, both the dorsal premotor cortex (dPMC) and the primary motor cortex (M1) were modulated by the relationship between the type of grasp that was adopted and the size of the stimulus.

**Conclusions/Significance:**

The demonstration that activity within the hAIPS is modulated according to different types of grasp, together with the evidence in humans that the dorsal premotor cortex is involved in grasp planning and execution offers a substantial contribution to the current debate about the neural substrates of visuomotor grasp in humans.

## Introduction

The highly developed ability of the hand to grasp and manipulate objects under precise visual control is one of the key features of the human motor system. The skilled use of the hand is fundamental to the technological, social and cultural progress of the human species [1]–[3]. The study of grasping was advanced by Napier's landmark work on precision and power grips [3]. According to Napier [3] there are only two main prehensile patterns, namely precision and power grips. The power grip (termed here as whole hand grasp; WHG) is a palmar opposition grasp in which all digits are flexed around the object to provide high stability. The precision grip (PG) has developed in primates for the manipulation of small objects with the tips of the thumb and fingers.

In recent years, there have been significant advances in our understanding of the neural mechanisms underlying the transformation of visual information about an object in the outside world into motor commands that allow the hand to be shaped for efficient grasp of the object. The huge variation in the shape, size and texture of the objects we must daily interact in a skillful and precise manner demands that this transformation provides a highly specific and selective matching of the object's properties to the motor commands for grasp and manipulation.

An important step forward in understanding how the brain controls grasp comes from the studies in which single neurons were recorded during naturalistic reach-to-grasp actions [4]–[8]. These studies showed that in macaques, grasp-related sensorimotor transformations are accomplished in a circuit connecting the anterior-most region within the lateral bank of the intraparietal sulcus (area AIP) with the ventral premotor area F5. It is postulated that AIP may furnish area F5 with visual signals of objects to aid in the selection of grasp configurations that are appropriate for their intrinsic attributes (e.g., size). The AIP-F5 network can then use the physical object properties to select the suitable motor schema according to the goal of the action [9].

An important feature of this network is that different neuronal populations code for specific types of hand shaping such as WHG and PG-the most represented type-characterized by the opposition of the thumb to the index finger. Furthermore there is specificity for different finger configurations, even within the same grip type [4].

Many neuroimaging studies have explored in humans the existence of a cortical grasping circuit similar to that described in monkeys [see 10], [11 for a review] revealing activation within the putative homolog of macaque areas AIP and F5 [12], [16]–[18], [19]–[21]. However, although they contribute noticeably to our understanding of the neural circuit underlying grasping in humans, they leave open the question of whether such circuit in humans has a special role in the coding of grasp type. Some studies varied the size and shape of the objects, but subjects were requested to reach towards and grasp the object by using a PG in all cases [12]–[15]. Other studies, asked subjects to perform non-visually-guided isometric grip tasks [16]–[18] which were not comparable to the above mentioned studies and to the reach-to-grasp tasks used with monkeys. One study in our lab considered reach to grasp movements towards objects differing in size, and subjects were not instructed on how to grasp the object [19]. This brought to the execution of a natural PG movements for small objects and a natural WHG for large objects. Significant activity was detected within hAIP for PG but not for WHG movements. Although suggestive of differential activity within a key grasping area depending on the type of performed grasp, the different pattern of activation for the two types of grasp could have arisen from the different size of the stimuli and not from the diverse posture assumed by the hand. Indeed, physiological studies have reported a subset of neurons within AIP that respond to the visual presentation of 3D objects in the absence of action [5]–[7]. The critical manipulation appears to be the use of the same object while instructing the subjects to use different grips.

**Figure 1:**
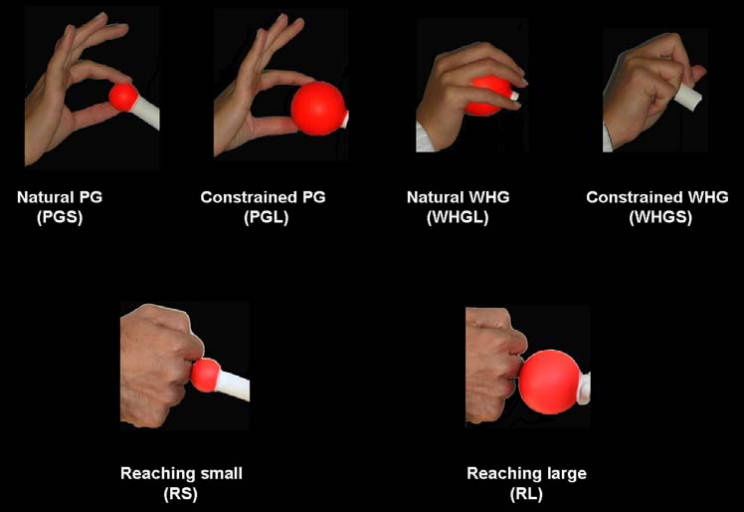
Stimuli and experimental design. Subjects viewed one of the two stimuli and performed three different tasks. In the PG tasks (PGS and PGL), they grasped the stimulus with a PG; in the WHG tasks (WHGL and WHGS), they grasped the stimulus with a WHG; in the reaching tasks (RS and RL), they touched the stimulus the knuckles, with the hand closed like in a fist. Subjects were informed about the movement to perform (PG, WHG or reaching) with a sound delivered through headphones. All actions had to be performed with the right hand. Stimulus dimension was randomized across and within subjects.

Therefore, we studied the kinematics and the fMRI activation pattern in right handed humans during the performance of a reach-to-grasp movement towards stimuli affording different types of grasp in ‘natural’ and ‘constrained’ conditions. For the natural grasp conditions subjects used a PG for the small stimulus and a WHG for a large stimulus. These conditions were termed respectively “PGS” and “WHGL” (Fig. 1). These natural conditions were compared with ‘constrained’ grasp conditions in which, irrespective of the size of the stimulus, the subject was instructed to consistently use either a PG or a WHG. These conditions were named “WHGS” and “PGL”, respectively (Fig. 1).

We also performed a kinematic experiment to examine whether the stimuli used for the fMRI experiment were able to elicit differential kinematic patterning for PG and WHG [22], and whether such a pattern was modified when the subjects were constrained in the use of a type of grasp which was incongruent with respect to the to-be-grasped stimulus.

We took advantage of evidence from single unit recordings in monkeys [4], [23] to address two critical questions: (i) whether varying hand conformation within the same class of grasp according to different types of grasp (e.g., PG and WHG) requires similar visuomotor transformations; and (ii) whether central mechanisms for the guidance of grasping are sensitive either to object size, type of grasp or the match between type of grasp and stimulus size (e.g., WHGS). Although our previous study [19] suggested that only precise grasping movements directed towards small stimuli significantly drive activity within hAIP, it is quite possible that hAIP may be also specialized for 3D object processing, regardless the nature of the task and/or that hAIP may be specialized for precise grasping movements independently from stimulus size. Therefore comparing brain activity for natural and constrained grasps towards the same stimulus provides an ideal opportunity to understand the functional contribution of relevant key areas for grasping.

## Materials and Methods

### Functional MRI

#### Subjects

Nineteen healthy subjects (12 female and 7 male; age range: 19–30 years) participated in the experiment. They were all right-handed as measured by the Edinburgh Handedness Inventory [24] and they had no neurological or psychiatric history, or any motor pathology; vision acuity was normal or corrected-to-normal. All gave informed written consent before entering the scanner room. The study was approved by the University of Padua Ethics Committee. Three subjects were not included in the analysis due to the presence of strong head motion.

#### Apparatus

The goal-directed actions were performed by the use of a grasping apparatus, a metal-free rotating table mounted on a plexiglass structure which allows the presentation of real 3D stimuli. The grasping apparatus could be rotated to each of the two faces, upon which the stimuli were attached, between trials. The experiment was conducted within an illuminated room. During the experiment, the subjects lay supine within the magnet with the head tilted at an angle (∼30 deg) and supported with a foam wedge, that permitted direct viewing of the stimuli without mirrors. Such direct viewing avoids introducing additional transformations required by mirror-viewing [14], [25]. The grasping apparatus was placed approximately 15 cm above the subject's pelvis in order to present the stimuli at a comfortable and natural grasping distance without the need for shoulder movements. In order to maintain constant the hand starting position constant across subjects and trials subjects wore a metal-free belt upon which a pad was attached. The hand was in a relaxed position laying with the palm upon the pad.

#### Stimuli

The stimuli consisted of two spherical plastic objects of different dimensions (small stimulus: 3 cm diameter; large stimulus: 6 cm diameter). We used a regular geometric shape rather than functional objects (i) for comparability with macaque neurophysiology studies [26], [27] and (ii) to examine grasping in a general manner rather than the left-hemisphere network specialized for functional objects such as tools [28]. Care was taken to chose a stimulus dimension which elicited two different types of grasp: PG and WHG. We confined our investigation to these two types of grasp for two main reasons: (i) according to Napier [3], [29] PG and WHG have to be considered as the two main types of grasp from which other grasps can be derived; (ii) neurophysiogical studies have clearly identified distinct neuronal populations subserving these two types of grasp [4]. All subjects naturally adopted a PG to grasp the small stimulus and a WHG to grasp the large stimulus.

#### Task procedures

Subjects were requested to perform three different actions towards either the small or the large stimulus (Fig. 1): (i) grasping the stimulus independently from its size with a PG; (ii) grasping the stimulus independently from its size with a WHG; (iii) reaching the stimulus and touch it with the knuckles of the hand, maintaining it in a closed fist (the fist posture was similar for both small and large objects) (Fig. 1). This type of reaching action was chosen as to minimize distal involvement. Subjects were instructed to unfold the action at a natural speed and were informed about the type of movement to perform through a sound delivered by pneumatic MR-compatible headphones: (i) PG -low tone (duration: 200 ms; frequency: 1,7 kHz); (ii) WHG-high tone (duration: 200 ms; frequency: 210 Hz:); Reaching-a double tone was delivered (two tones of 70 ms duration: 445Hz, staggered by a 60 ms silence). Subjects were specifically instructed to start their action toward the stimulus only when the sound was delivered.

From the control cabin beside the scanner room it was possible to monitor the person inside the scanner through a glass. Therefore it was possible to control whether the subjects responded to the sounds and whether they were performing the action corresponding to the presented tone. Trials in which subjects did not grasp or reach the object appropriately and/or the movement started before the presentation of the sound were discarded and they were not included in the analysis.

#### Experimental design

The experiment was conducted by using an event-related design. Inter Stimulus Interval (ISI) varied from 3 to 8 seconds with a ‘long exponential’ probability distribution [30]. ISIs distribution was fully randomized across trials in each run for each subject. Action towards the stimulus (PG, WHG or reaching) and stimulus dimension (small or large) were manipulated as to create six different conditions (Fig. 1): 1, “Natural PG towards the small object” (PGS); 2, “Constrained PG towards a large object” (PGL); 3, “Natural WHG towards a large object” (WHGL); 4) “Constrained WHG towards a small object (WHGS); 5)“Reaching towards a small object” (RS); 6) “Reaching towards a large object” (RL). A total of 360 trials was administered (60 trials per condition) in a randomized order. Trials were divided in 4 runs of 90 trials each, with a short rest between runs.

#### Imaging parameters

Images were acquired with a whole-body 3 T scanner (Siemens Magnetom Trio, TIM system) equipped with a standard Siemens 12 channels coil. Functional images were acquired with a gradient-echo, echo-planar (EPI) T2*-weighted sequence in order to measure blood oxygenation level-dependent (BOLD) contrast throughout the whole brain (47 contiguous axial slices acquired with descending interleaved sequence, 64×64 voxels, 3.3×3.3×3 mm resolution, FOV = 210×210 mm^2^, flip angle = 90°, TE = 30 ms). Volumes were acquired continuously with a repetition time (TR) of 3 s; 117 volumes were collected in each single scanning run (5:51 minutes; 4 scanning runs in total). High-resolution T1-weighted images were acquired for each subject (3D MP-RAGE, 176 axial slices, data matrix 256×256, 1 mm isotropic voxels, TR = 1859 ms, TE = 3.14 ms, flip angle = 22°).

#### Data analysis

Data analysis was performed using the software package SPM5 (Wellcome Department of Imaging Neuroscience, University College of London, UK-http://www.fil.ion.ucl.ac.uk/spm). The first four scans for each session were excluded from data analysis because of the non-equilibrium state of magnetization. For each subject, images underwent motion correction and unwarping, and each volume was realigned to the first volume in the series. The mean of all functional images was then co-registered to the anatomical scan, previously corrected for intensity inhomogeneities. EPI images were then normalized adopting the MNI152 template, supplied by the Montreal Neurological Institute (http://www.mni.mcgill.ca/) and distributed with the software SPM. Finally, images were smoothed using a 6.6×6.6×6 mm FWHM 3D Gaussian kernel (twice the native voxel size). High-pass filtering was also applied to remove low-frequency drifts in signal.

At the first level, for each single subject the different types of action corresponding to the six experimental conditions (PGS, PGL, WHGL, WHGS, RS and RL, see Fig. 1) were modelled as separate event types (duration: 2 s). Regressors were defined on the timing of presentation of each experimental condition, and these functions were convolved with a canonical, synthetic HRF (haemodynamic response function) and its first-order temporal derivative in order to produce the individual models [31]. Errors (incorrect actions) were modelled as a seventh condition of no interest. For each subject, all regressors were incorporated into General Linear Models [GLM–32], and motion correction parameters created during the realignment stage, were included in the analysis as a covariate of no interest. This was done in order to model residual effects due to head motion. Individual models were separately estimated and contrasts were defined in order to pick out the main effects of each experimental condition. Then for each subject the reaching related activation was subtracted from the correspondent reach-to-grasp related activation. This procedure, which has been adopted in several previous neuroimaging studies on grasping [13], [15] allows for the detection and isolation of activations confined to hand shaping. The subtraction was applied to all four grasping conditions (PGS-RS; PGL-RL; WHGS-RS; WHGL-RL) for each subject and the resulting contrasts were then entered into a second level analysis in which subjects served as a random effect. The resulting SPM*{t}* maps reflected areas in which variance related to the experimental manipulation was captured by the HRF adopted in the GLM. Clusters were reported only if surviving a threshold of p<.05 (FWE-corrected for multiple comparisons). Coordinates of the resulting significant activations were converted to the Talairach reference space [33] using the nonlinear transformation procedure developed by Dr. Matthew Brett (mni2tal, available at http.//www.mrc-cbu.cam.ac.uk/Imaging/Common). To localize activations we used the Talairach Daemon database implemented in the brain atlas developed by the Neurology University Hospital of Muenster (available at http://www.neuro03.unimuenster.de/ger/t2tconv/index.html) and the Duvernoy atlas [34]. Further, the SPM Anatomy Toolbox [35] based on three-dimensional probabilistic cytoarchitectonic maps was used to determine the probability associated with activity peaks revealed by the random effects analysis: all the detected activations were associated with a probability value equal or greater than 50% within the respective cytoarchitectonic map (motor, premotor and somatosensory cortices) [36]–[37].

## Results

To test our specific experimental hypotheses we performed three planned contrasts: (i) in order to assess whether there was a differential level of activity depending on the size of the stimulus we compared activity for the ‘small’ stimulus versus activity for the ‘large’ stimulus independently from type of grasp; (ii) in order to explore whether there was a differential level of activity depending on grasp type we compared activity for PG versus activity for WHG independently from stimulus size; and (iii) to ascertain the level of congruency between the stimulus and the grasping schema produced differential activation patterns, brain activity for natural and constrained grasps was compared.

### Activity related to object size

The contrast comparing activity for the small sized object with activity for the large sized object independently from type of grasp [(PGS+WHGS)>(PGL+WHGL)] did not reveal any significant difference in activity. Similarly, the opposite contrast comparing activity for the large sized object with activity for the small sized object independently from type of grasp [(PGL+WHGL)>(PGS+WHGS)] lead to non-significant results. Therefore the hypothesis that the ‘size’ computation may account for the differential activations within key areas concerned with visuomotor grasping can be ruled out.

### Activity related to different types of grasp

**Figure 2:**
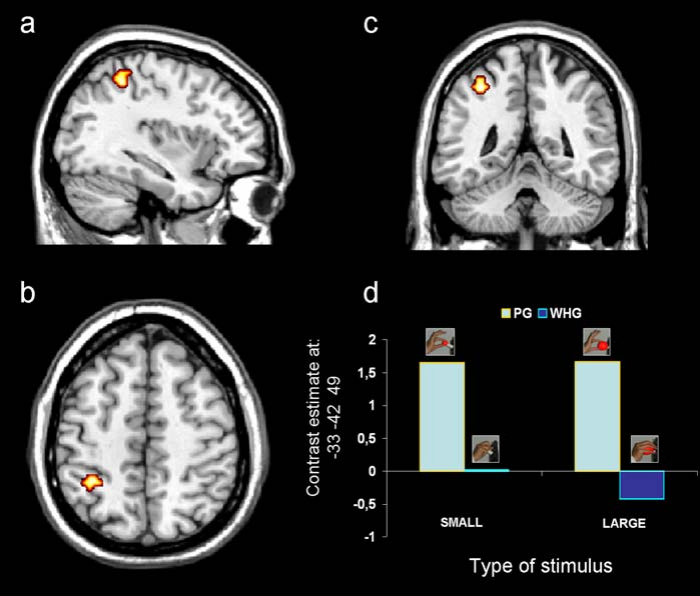
Group statistical map for the contrast comparing type of grasp (PG vs WHG). The contrast revealed difference of activity only within the left aIPS (*p*<0.05, FWE corrected). The group statistical map is superimposed on the canonical brain of the MNI series in sagittal (a) axial (b), and coronal (c) sections. (d) This panel shows contrast estimate. Talairach coordinates of areas in which the level of activity significantly differed between conditions are reported in Table 1.

The contrast comparing PG with WHG [(PGS+PGL)>(WHGS+WHGL)] revealed a significant difference in activity located in the left anterior part of the intraparietal sulcus (hAIP; Fig. 2 and Table 1) for PG, but not for WHG. We located the focus of activation at the junction of the aIPS and the postcentral sulcus (PCS) in the left hemisphere of all 19 subjects. The opposite comparison, contrasting activity for WHG with activity for PG [(WHGS+WHGL)>(PGS+PGL)] did not lead to any significant result. Hence, the hypothesis that hAIP activity modulates with respect to grasp type was supported.

**Table 1:** Brain regions showing significant differences in activation for the contrasts comparing precision grip versus whole hand grasp (PGS+PGL)>(WHGS+WHGL) and natural versus constrained grasps (PGL+WHGS)>(PGS+WHGL).

Contrast	Side	Area	BA	k	p (cluster level)	p (voxel level)	t	x	y	z
**(PGS+PGL)>(WHGS+WHGL)**	L	**aIPS**	**40**	**19**	**.000**	**.001**	**6.27**	**−33**	**−42**	**49**
**(PGL+WHGS)>(PGS+WHGL)**	**L**	**Pre-central gyrus**	**4**	**142**	**.000**	**.000**	**9.18**	**−36**	**−20**	**48**
	L	Pre-central gyrus	6			.000	6.58	−40	−14	59
	L	Post-central gyrus	3			.002	6.13	−40	−22	59
	**R**	**Post-central gyrus**	**3**	**14**	**.000**	**.000**	**6.93**	**52**	**−20**	**40**
	**R**	**Pre-central gyrus**	**6**	**10**	**.002**	**.003**	**5.99**	**40**	**−10**	**54**

For each local maxima brain structure, Brodmann area (BA), number of activated voxels (k), Talairach coordinates and statistical significance

(p<0.05 FWE corrected) for t-tests comparisons are reported (for both cluster-and voxel-level). L = Left; R = Right; cluster size: ≥10. All coordinates fall within the central area (probability: 50–100%) of the probabilistic cytoarchitectonic maps of reference (Eickoff et al., 2005).

### Activity related to natural versus constrained grasps

**Figure 3:**
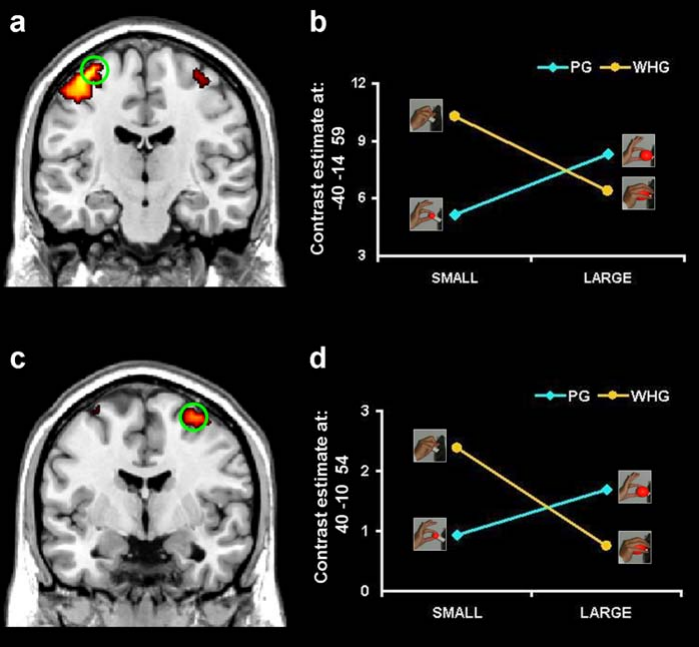
Group statistical map for the contrast comparing natural and constrained grasp: dPMC activation. The contrast revealed difference of activity within the dPMC bilaterally (*p*<0.05, FWE corrected). The group statistical map is superimposed on the canonical brain of the MNI series in sagittal (a), axial (b), and coronal (c) sections. d) This panel shows contrast estimate. Green circles indicate brain areas whose level of activity was significant between conditions. Talairach coordinates for these areas are reported in Table 1.

**Figure 4:**
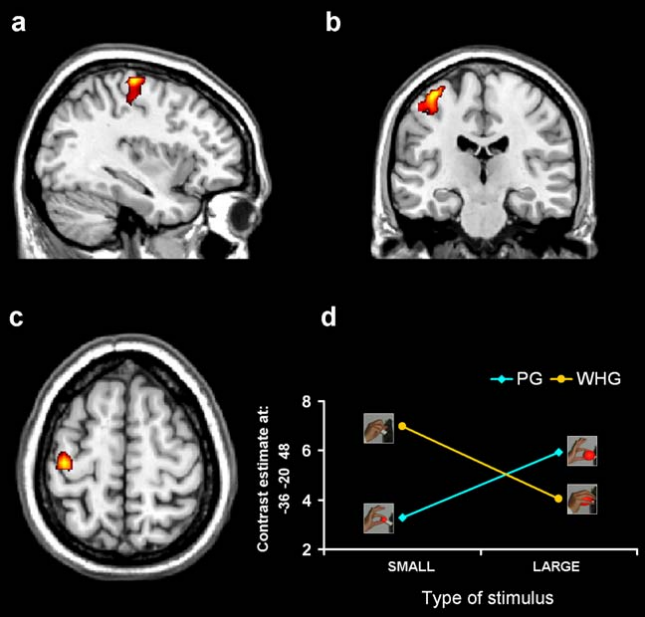
Group statistical map for the contrast comparing natural and constrained grasp: M1 activation. The contrast revealed differential activation within the left M1 (*p*<0.05, FWE corrected). The group statistical map is superimposed on the canonical brain of the MNI series in sagittal (a), axial (b), and coronal (c) sections. d) this panel shows contrast estimate. Talairach coordinates for areas in which the level of activity significantly differed between conditions are reported in Table 1.

The comparison of constrained versus natural grasps [(PGL+WHGS)>(PGS+WHGL)] showed a significant difference in activity within the dorsal premotor cortex (dPMC; Fig. 3 and Table 1) bilaterally and the left pre-central gyrus corresponding to the primary motor cortex (M1; Fig. 4 and Table 1). The opposite comparison for both areas, [(PGS+WHGL)>(PGL+WHGS)] did not lead to any significant result. Thus, in line with our prediction, asking subjects to grasp the same object with different types of grasp allows to uncover differential levels of activity within key-areas involved in visuomotor grasping.

### Behavioural Experiment

#### Subjects

Twelve subjects (4 men, 8 women; aged 20–25 years) volunteered to participate. All showed right-handed dominance [24] and were naïve as to the experimental design or purpose. None reported visual or psychomotor dysfunction.

#### Stimuli, apparatus and procedure

The stimuli and the procedures were similar in all respects to those described for the fMRI experiment. Infrared reflective markers (0.25 mm diameter) were taped to the following points on the subjects' right upper limb: (1) wrist–dorsodistal aspect of the radial styloid process; (2) thumb – ulnar side of the nail; and (3) index finger–radial side of the nail. Markers were fastened using double-sided tape. Movements were recorded using an ELITE motion analysis system (Bioengineering Technology & Systems [B|T|S]). Four infrared cameras (sampling rate 100 Hz) placed 120 cm away from each of the four corners of the table captured the movement of markers in 3D space. Co-ordinates of the markers were reconstructed with an accuracy of 0.2 mm over the field of view. The standard deviation of the reconstruction error was 0.2 mm for the vertical (Y) axis and 0.3 mm for the two horizontal (X and Z) axes. The experimenter was given on-line computer screen feedback of the three-dimensional position of each marker–if one marker was missing during task performance the trial was manually discarded. Experimentation continued until the required number of successful trials was collected. A block of trials (N = 10) for each experimental condition (PGS, PGL, WHGL, WHGS) was administered.

#### Data processing

In order to ascertain possible differences at the level of movement planning, initiation time was calculated as the time between the presentation of the tone and the release of a switch embedded within the hand starting location. The ELIGRASP software package (B|T|S|) was used to analyze the data and provide a 3-D reconstruction of the marker positions as a function of time. The data were then filtered using a finite impulse response linear filter (transition band = 1 Hz, sharpening variable = 2, cutoff frequency = 10 Hz). Following this operation, the tangential speed data for the wrist marker were used to determine the onset of the movement using a standard algorithm (threshold for movement onset was ∼5 cm/s). Movement onset was taken as the earliest time at which movement of the wrist occurred. Movement offset was taken at the latest time at which the movement of the thumb and index finger occurred. As for the fMRI experiment the analysis was confined to the grasp component. Specifically, only the dependent variables which have demonstrated robust ‘type of grasp’ effects in previous research [e.g., 10, 22] are considered. (i) the amplitude of maximum grip aperture (the maximum distance between the thumb and index finger); (ii) the time of maximum peak grip aperture. Further, to evaluate the degree of accuracy at end grasp, the grasp angle variability (standard deviations of the angle between the index finger and thumb markers at the end of the grasp) was computed. This latter parameter was calculated only for PGS and PGL conditions given that the configuration assumed by the hand for WHGL and WHGS did not allow for a precise determination of such measure.

The mean value of each measure for each subject was analysed with an Analyses of Variance (ANOVA; 0.05 alpha level of significance). The within-subjects factors were type of grasp (PG, WHG), and stimulus size (large, small). Bonferroni corrections were applied to the contrasts of interest (throughout the text significant values are indicated). Preliminary analyses were conducted to check for normality, univariate and multivariate outliers, with no serious violations noted.

## Results

The main factor type of grasp was significant for movement time [F(1,11) = 25.83, p<0.001; η^2^_p_ = 0.69] and the amplitude of maximum grip aperture [F(1,11) = 32.06, p<0.0001; η^2^_p_ = 0.81]. Specifically, movement time was longer (743 vs 658 ms) and the amplitude of maximum grip aperture was smaller (78 vs 110 mm) for PG than for the WHG. These results indicate that the chosen stimuli elicited differential kinematic patterns as previously reported [22], [38]–[39].

**Figure 5:**
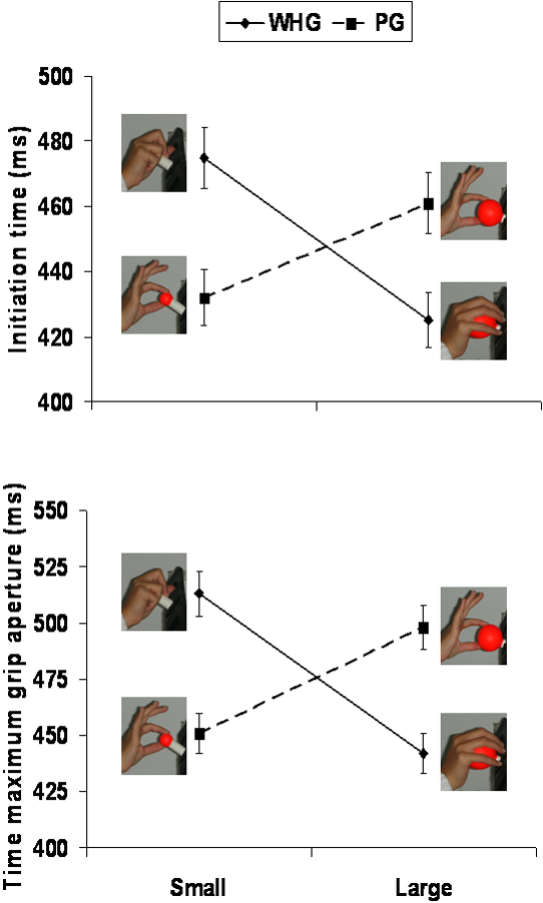
Graphical representation of the interaction type of stimulus by type of grasp for initiation time and the time of maximum grip aperture. a) The interaction between type of stimulus and type of grasp indicate an increase in initiation time for constrained grasps with respect to natural grasps. b) The interaction between type of stimulus and type of grasp indicate that the time of maximum grip aperture was anticipated for constrained grasps with respect to natural grasps. Dotted lines refer to natural and constrained grasps towards the small stimulus. Solid lines refer to natural and constrained grasps towards the large stimulus.

**Figure 6:**
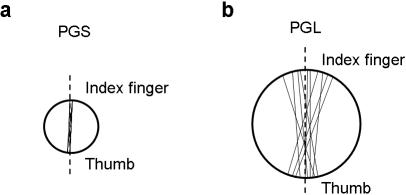
Grasp angle for natural and constrained precision grip tasks. a) Pattern of grasp angle for a precision grip movement performed towards the small stimulus (Natural conditions). Please note the consistency of contact points for the index finger and the thumb. b) Pattern of grasp angle for a precision grip movement performed towards the large stimulus (Constrained conditions) Please note that for this task variability for the index finger and the thumb contact points increases.

The two way interaction stimulus size by type of grasp was significant for initiation time [F(1,11) = 21.31, p<0.0001; η^2^_p_ = 0.85] and the time of maximum grip aperture [F(1,11) = 18.04, p<0.0001; η^2^_p_ = 0.79]. As shown in Figure 5 initiation time was longer and the time of maximum grip aperture anticipated for constrained (WHGS and PGL) than for natural grasps (WHGL and PGS) (p_s_<0.05). For the measure ‘grasp angle variability’ *t* test analysis revealed that variability was higher for the incongruent PGL than for the congruent PGS (p<0.0001; Fig. 6).

## Discussion

The goal of the present study was to investigate whether key areas within the visuomotor circuit underlying grasping are differently alerted depending on grasp configurations and whether activity within these areas is modulated by the congruency between the perceptual features of the to-be-grasped object and the type of performed grasp. This was done by using both fMRI and kinematic methods. Here we report evidence that activity within hAIPS varied depending on grasp configurations, whereas activity within M1 and the dPMC varied depending on the level of congruency between the planned grasp and the to-be-grasped stimulus.

### Activity related to different types of grasp

As reported here, several functional neuroimaging studies have indicated that focal activation within the hAIP of the healthy brain occurs in association with visually guided grasping [12]–[15], [20]. Although in previous studies the size and shape of the objects was varied, subjects were requested to use a PG in all cases [12]–[15]. Those that did ask subjects to perform a specific type of grasp (i.e., PG or WHG), related to the size of the to-be-grasped object, considered only non-visually-guided isometric grip tasks [16]–[17] or did not report separate data for different types of grasp [16]. Therefore whether hAIP has a special role in the coding for specific types of hand shaping during reaching is essentially unknown.

Our findings add to this literature by showing that a significantly greater level of activity in hAIP was found for PG than for WHG tasks. This result shows that in humans, as in macaque, activity within this area is tuned to type of grasp. Thus, in humans as in monkeys, AIP has come to be viewed as a prototypic region subserving various forms of grasp formation [5]–[7]. This conclusion is bolstered by the result that hAIP did not activate significantly with respect to object size. Thus, the different pattern of activation for the two types of grasp could not have arisen from the different size of the stimuli but from the diverse configuration assumed by the hand.

The higher level of activation in hAIP, together with the observed kinematic pattern reflects the need for additional sensory-motor control mechanisms for PG. This result is in line with previous neurophysiological and behavioural reports [24], [38]. In first instance, inactivation studies in which the monkeys were injected with a GABA-agonist (muscimol) in AIP [26] revealed a clear impairment of the grasping behavior of the hand contralateral to the inactivated hemisphere, with PG movements being the most impaired. In second instance, precise placement of two digits upon the surface area and the force during pulp-to-pulp opposition engaging pairs of individual digits might be more demanding in terms of neural control, especially when the size of the grasped object would require more than two fingers for a stable prehension (as for the PGL condition) [38] and subsequent manipulation. In this respect the present kinematic results confirm this view: the time of maximum grip aperture was reached earlier for PG than for WHG. This suggests that for a PG more time was needed during the grasp closing phase as to determine more precise contact points for subsequent manipulation. Therefore on the basis of both kinematics and fMRI evidence it may be tenable that the difference in activation between PG and WHG reflects the need for additional sensory-motor control mechanisms for PG and that hAIP may play a critical role in this behaviour [15].

### Activity related to natural versus constrained grasps

Here we report evidence that the imposition of one grasp type for both small and large objects resulted in mismatching of appropriate grasp to object size which was evident at both behavioral and neural level. In behavioral terms, the increase in initiation time for constrained grasps suggest that the planning of a precise grasp for a large diameter object not only infers inhibition of neural processes for a whole hand grasp, but also activation of patterning for a large aperture. Similarly, use of a whole hand grasp for a small diameter object not only infers inhibition of neural processes for a precise grasp, but also activation of patterning for a small aperture. In other words, increasing the time to initiate the movement may indicate that some sort of interference may arose during the planning of unnatural grasps. Further, kinematic analysis suggests that such possible interference effects carry over during action execution. The anticipation of maximum grip aperture signifies that more time is needed to close the hand upon the object. The increase in end-point variability indicates that it might be more difficult to establish an appropriate placement of two digits upon a larger surface area.

In neural terms, the dPMC and M1 were mostly alerted by the mismatch between stimulus size and the type of adopted grasp. The dPMC has been extensively studied during the last two decades. A number of studies have demonstrated that the set- and movement-related discharge of the dorsal premotor neurons is correlated to parameters of reaching movements such as direction and amplitude [40]–[43]. In these studies, however, only proximal forelimb movements were taken into account, the contribution of the distal forelimb movements to the neuronal discharge not being considered until recently. Raos [44] demonstrated that within the dPMC (area F2) a distal forelimb field also exists. Finger movements can be evoked by intracortical microstimulation in this field. Furthermore, single-neuron recording revealed the presence in this area of many neurons related to distal actions [44]. The properties of these neurons have been investigated by use of a behavioral paradigm that allows the study of neuronal discharge during grasping of different 3D objects [45]. This study provides compelling evidence that in the distal forelimb representation of area F2 there are neurons that are selective for the type of prehension required for grasping the object. These results indicate an important role of the dPMC in the control of goal-related hand movements. It was the first demonstration that neurons within the dPMC are also involved in grasping execution. The activity of these grasping neurons was not related to individual finger movements, but to the grasping action as a whole. Specifically, the proposal here is that area F2 grasping neurons has the role of keeping in memory the motor representation of the object and combine it with visual information as to continuously update the configuration and orientation of the hand as it approaches the object to be grasped. In this view the dPMC involvement during goal-directed actions appears to be highly correlated with the accuracy requirement of the ongoing movement [46]. The timing of this correlations suggest that accuracy information is available for movement planning and on-line monitoring.

In humans the contribution of the dPMC to hand movements, the time course of its involvement and its hemispheric dominance is essentially unknown. Therefore the present results shed new light on the functional mechanisms presiding over the control of visually guided hand-grasping actions in humans. The increase of activity within the dPMC for constrained grasps may provide the evidence that in humans as in monkeys this area is involved in the control of grasping. In order to resolve the mismatch between type of grasp and stimulus size which occurs for the constrained conditions this area shows an increase of activation which might be necessary as to provide the necessary control. In this respect, we demonstrate using functional neuroimaging that the dPMC may play a crucial role in monitoring the configuration of fingers during planning and execution of specific grasping actions. In this respect the present findings add to what has been recently reported by Davare and colleagues [47]. By means of TMS they produced a transient virtual lesion of the dPMC in both hemispheres while a subject performed a precision grip-lift task with their right hand. It was found that a virtual lesion of the left dPMC impaired the proper coupling between the grasping and the lifting of an object. Hence, our result of an increase of activity within the dPMC for constrained grasps may not only be indicative of an involvement of this areas for the planning and the on-line monitoring of grasping actions, but also that in providing the necessary control as to correct the timing of the lifting phase with respect to the grasping phase for an unnatural grasp. Indeed our constrained conditions elicited an awkward finger positioning which might have prevented the establishment of suitable contact points for lifting. This hypothesis is in line with the present kinematic analysis of maximum grip aperture and contact points. Importantly, the pattern of the significant interaction for the time of maximum grip aperture exactly corresponds to the pattern of activation observed for the dPMC. To an anticipation of the time of maximum grip aperture and an increase in grasp angle variability corresponded grater activity for constrained than for natural grasps. This indicates that in order to prolong the grasp closing phase-dictated by a greater difficulty for the determination of suitable contact points-the dPMC had to increase the activity level.

A further issue is concerned with the fact that a significant activity increase within the dPMC was found for both hemispheres. Bilateral premotor activity has been previously reported for the ventral premotor cortex (vPMC) in relation to “motor errors” [48] and to motor response competition [49]. In this respect evidence from neurophysiology indicate that the ventral premotor region F5 is reciprocally connected with a sector of the dorsal premotor cortex, area F2vr, where grasping neurons are located [44] . Importantly, area F2vr contains visuomotor neurons similar to the corresponding F5 neurons. Therefore, it might well be that given the reciprocal connections also the dPMC shows a similar activation pattern. When ‘errors’ are concerned with grasping and more specifically when these errors needs to be adapted on-line both hemispheres contribute to such process. Given that grasping a large object with only two fingers (PGL) or the small object with the whole hand (WHGS) are unusual ways to interact with such objects, it can be hypothesized that those movements are interpreted as a sort of “motor error”. Along these lines, we suggest that the dPMC bilateral activation for incongruent prehension trials might reflect a processing concerned with the on-line monitoring of a movement error which may prevent the completion of the action goal.

A pattern of activity similar to that observed for the dPMC was also found for M1. A number of neurophysiological studies have emphasized the extensixe M1 activity that occurs during performance of grasping tasks [50]–[52], including the demonstration that M1 neurons show remarkably different activity during performance of different grip [27], [53]–[56]. However, what is so far lacking in the human neuroimaging literature on cortical control of grasping is a systematic documentation of neuronal activity in M1 during performance of different types of grasp. Here we demonstrate that our experimental manipulation was able to reveal that activity in M1 was modulated by the level of congruence between type of grasp and stimulus size. The similar pattern of activation for the dPMC and M1 confirm previous neurophysiological evidence suggesting that F2 may control the execution of grasping actions through their direct connections with M1 [45]. In keeping with these lines of evidence it might well be that the dPMC representation of stimulus size-specific grasp described in the present study is transformed within M1 to recruit motor outputs to the hand that can modify hand shape appropriate for successful grasp and manipulation of the object [27].

The pattern of the significant interaction for the time of maximum grip aperture mirrors exactly the pattern of activation observed for the dPMC and M1. To an anticipation of the time of maximum grip aperture and an increase in grasp angle variability corresponded greater activity for constrained than for natural grasps. This indicates that in order to guide an unnatural grasping, and to determine suitable contact points, both the dPMC and M1 had to increase the activity level.

### Conclusions

The present results shed new light on the functional mechanisms presiding over the control of visually guided hand grasping actions. Specifically, the strength and the novelty of our findings comes chiefly from the natural versus constrained contrast enabling us to better define the functional properties of key areas involved in the control of grasping. Crucially, they extend the current human neuroimaging literature by strengthening the human/monkey homology in two important ways. First, they provide neuroimaging support to many neurophysiological results showing that in monkeys AIP, the dPMC and M1 there is a category of motor neurons that represent either the goal of the action and the way in which the action is executed. Second, they highlight the crucial role played by the dPMC in monitoring the configuration of fingers during planning and execution of reach-to-grasp actions. Taken together these results provide new evidence for the existence of a visuomotor grasping circuit in humans similar to that revealed in monkeys which play a key role in hand preshaping.

## References

[1] Lemon RN (1993). The G. L. Brown Prize Lecture. Cortical control of the primate hand.. Exp Physiol.

[2] Tallis R (2004). The Hand. A Philosophical Enquiry into Human Being..

[3] Napier JRJ (1956). The prehensile movements of the human hand.. J Bone Joint Surg,.

[4] Rizzolatti G, Camarda L, Fogassi L, Gentilucci M, Luppino G (1988). Functional organization of inferior area 6 in the macaque monkey. II. Area F5 and the control of distal movements.. Exp Brain Res.

[5] Taira M, Mine S, Georgopoulos AP, Murata A, Sakata H (1990). Parietal cortex neurons of the monkey related to the visual guidance of hand movement.. Exp Brain Res.

[6] Sakata H, Taira M, Mine S, Murata A, Caminiti R, Johnson PB, Burnod Y (1992). Hand-movement related neurons of the posterior parietal cortex of the monkey: their role in visual guidance of hand movements.. Control of Arm Movement in Space: Neurophysiological and Computational Approaches.

[7] Murata A, Gallese V, Luppino G, Kaseda M, Sakata H (2000). Selectivity for the shape, size and orientation of objects for grasping in neurons of monkey parietal area AIP.. J Neurophysiol.

[8] Raos V, Umilta MA, Murata A, Fogassi L, Gallese V (2006). Functional properties of grasping-related neurons in the ventral premotor area F5 of the macaque monkey.. J Neurophysiol.

[9] Rizzolatti G, Luppino G (2001). The cortical motor system.. Neuron.

[10] Castiello U (2005). The neuroscience of grasping.. Nat Rev Neurosci.

[11] Culham JC, Cavina-Pratesi C, Singhal A (2006). The role of parietal cortex in visuomotor control: What have we learned from neuroimaging?. Neuropsychologia.

[12] Binkofski F, Dohle C, Posse S, Stephan KM, Hefter H (1998). Anterior intraparietal area subserves prehension.. Neurology.

[13] Culham JC, Danckert SL, DeSouza JF, Gati JS, Menon RS (2003). Visually guided grasping produces fMRI activation in dorsal but not ventral stream brain areas.. Exp Brain Res.

[14] Culham JC, Kanwisher N, Duncan J (2004). Human brain imaging reveals a parietal area specialized for grasping.. Attention and Performance XX: Functional Neuroimaging of Visual Cognition.

[15] Frey SH, Vinton D, Norlund R, Grafton ST (2005). Cortical topography of human anterior intraparietal cortex active during visually guided grasping.. Cogn Brain Res.

[16] Ehrsson HH, Fagergren A, Johnsson T, Westling G, Johansson RS (2000). Simultaneous movements of upper and lower limbs are coordinated by motor representations that are shared by both limbs: a PET Study.. Eur J Neurosci..

[17] Ehrsson HH, Fagergren E, Forssberg H (2001). Differential fronto-parietal activation depending on force used in a precision grip task: an fMRI Study.. Neurophysiol.

[18] Grèzes J, Armony L, Rowe J, Passingham RE (2003). Activations related to ‘mirror’ and ‘canonical’ neurons in the human brain: an fMRI Study.. Neuroimage.

[19] Begliomini C, Wall MB, Smith AT, Castiello U (2007). Differential cortical activity for precision versus whole-hand visually guided grasping.. Eur J Neurosci.

[20] Binkofski F, Buccino G, Posse S, Seitz RJ, Rizzolatti G (1999). A frontoparietal circuit for object manipulation in man: evidence from an fMRI-Study.. Eur J Neurosci.

[21] Kuhtz-Buschbeck JP, Ehrsson HH, Forssberg H (2001). Human brain activity in the control of fine static precision grip forces: an fMRI study.. Eur J Neurosci.

[22] Gentilucci M, Castiello U, Corradini ML, Scarpa M, Umiltà C (1991). Influence of different types of grasping on the transport component of prehension movements.. Neuropsychologia,.

[23] Murata A, Fadiga L, Fogassi L, Gallese V, Raos V, Rizzolatti G (1997). Object representation in the ventral premotor cortex (area F5) of the monkey.. J Neurophysiol.

[24] Oldfield RC (1971). The assessment and analysis of handedness: the Edinburgh Inventory.. Neuropsychologia.

[25] Cavina-Pratesi C, Goodale M, Culham JC (2007). FMRI reveals a dissociation between grasping and perceiving the size of real 3D objects.. PLoS ONE.

[26] Gallese V, Murata A, Kaseda M, Nikim N, Sakata H (1994). Deficit of hand preshaping after muscimol injection in monkey parietal cortex.. Neuroreport,.

[27] Umilta MA, Brochier T, Spinks RL, Lemon RN (2007). Simultaneous recording of macaque premotor and primary motor cortex neuronal populations reveals different functional contributions to visuomotor grasp.. J Neurophysiol.

[28] Johnson-Frey SH, Newman-Norlund R, Grafton ST (2005). A distributed left hemisphere network active during planning of everyday tool use skills.. Cereb Cortex.

[29] Napier J (1993). Hands..

[30] Hagberg GE, Zito G, Patria F, Sanes JN (2001). Improved detection of event-related functional MRI signals using probability functions.. Neuroimage.

[31] Henson RNA, Rugg MD, Friston KJ (2001). The choice of basis functions in event-related fMRI.. NeuroImage,.

[32] Friston KJ, Holmes AP, Worsley KJ, Poline JB, Frith C (1995b). Statistical Parametric Maps in Functional Imaging: A General Linear Approach.. Hum Brain Mapp.

[33] Talairach J, Tournoux P (1988). Co-Planar Stereotaxic Atlas of the Human Brain..

[34] Duvernoy P (1991). The Human Brain: Structure, Three-Dimensional Sectional Anatomy and MRI..

[35] Eickhoff SB, Stephan HE, Mohlberg H, Grefkes C, Fink GR (2005). A new SPM toolbox for combining cytoarchitectonic maps and functional imaging data.. Neuroimage.

[36] Geyer S, Ledberg A, Schleicher A, Kinomura S, Schormann T (1996). Two different areas within the primary motor cortex of man.. Nature.

[37] Grefkes C, Geyer S, Schormann T, Roland P, Zilles K (2001). Human somatosensory area 2: observer-independent cytoarchitectonic mapping, interindividual variability, and population map.. Neuroimage.

[38] Castiello U, Bennett KMB, Stelmach GE (1993). Reach to grasp: the natural response to a perturbation of object size.. Exp Brain Res,.

[39] Castiello U, Bonfiglioli C, Bennett KM (1996). How perceived object dimension influences prehension.. Neuroreport.

[40] Caminiti R, Johnson PB, Galli C, Ferraia S, Burnod Y (1991). Making arm movements within different parts of space: the premotor and motor cortical representation of a coordinate system for reaching to visual targets.. J Neurosci.

[41] Fu Q-G, Suarez JI, Ebner TJ Neuronal specification of direction and distance during reaching movements in the superior precentral premotor area and primary motor cortex of monkeys.. J Neurophysiol.

[42] Kalaska JF, Scott SH, Cisek P, Sergio LE (1997). Cortical control of reaching movements.. Curr Opin Neurobiol.

[43] Wise SP, Boussaoud D, Johnson PB, Caminiti R (1997). Premotor and parietal cortex: corticocortical connectivity and combinatorial computations.. Annu Rev Neurosci.

[44] Raos V, Franchi G, Gallese V, Fogassi L (2003). Somatotopic organization of the lateral part of area F2 (dorsal premotor cortex) of the macaque monkey.. J Neurophysiol.

[45] Raos V, Umilta MA, Gallese V, Fogassi L (2004). Functional properties of grasping-related neurons in the dorsal premotor area F2 of the macaque monkey.. J Neurophysiol.

[46] Gomez JE, Fu Q, Flament D, Ebner TJ (2000). Representation of accuracy in the dorsal premotor cortex.. Eur J Neurosci.

[47] Davare M, Andres M, Cosnard G, Thonnard JL, Olivier E (2006). Dissociating the role of ventral and dorsal premotor cortex in precision grasping.. J Neurosci.

[48] Manthey S, Schubotz RI, von Cramon DY (2003). Premotor cortex in observing erroneous action: an fMRI Study.. Brain Res Cogn Brain Res.

[49] Hazeltine E, Bunge SA, Scanlon MD, Gabrieli JD (2003). Material-dependent and material-independent selection processes in the frontal and parietal lobes: an event-related fMRI investigation of response competition.. Neuropsychologia.

[50] Hepp-Reymond MC, Seklis HD, Erwin J (1998). Functional organization of motor cortex and its participation in voluntary movements.. Comparative Primate Biology.

[51] Lemon RN (1981). Variety of functional organization within the monkey motor cortex.. J Physiol.

[52] Lemon RN, Hanby JA, Porter R (1976). Relationship between the activity of precentral neurons during active and passive movements in conscious monkeys.. Proc R Soc Lond B Biol Sci.

[53] Lemon RN, Mantel GW, Muir RB (1986). Corticospinal facilitation of hand muscles during voluntary movement in the conscious monkey.. J Physiol.

[54] Mason CR, Gomez JE, Ebner TJ (2002). Primary motor cortex neuronal discharge during reach-to-grasp: controlling the hand as a unit.. Arch Ital Biol.

[55] Morrow MM, Miller LE (2003). Prediction of muscle activity by populations of sequentially recorded primary motor cortex neurons.. J Neurophysiol.

[56] Muir RB, Lemon RN (1983). Corticospinal neurons with a special role in precision grip.. Brain Res.

